# An Overlooked Cause of Myocardial Infarction With Normal Coronaries Presenting as Stress Cardiomyopathy in Females

**DOI:** 10.7759/cureus.33251

**Published:** 2023-01-02

**Authors:** Annapoorna Singh, Krishna Patel, Femina Patel, John T Saxon, Anna Grodzinsky

**Affiliations:** 1 Internal Medicine, University of Kansas Health System St. Francis Hospital, Lawrence, USA; 2 Cardiology, Icahn School of Medicine at Mount Sinai, New York, USA; 3 Internal Medicine, University of California Riverside, Riverside, USA; 4 Cardiology, Saint Luke’s Mid America Heart Institute, Kansas City, USA

**Keywords:** takotsubo cardiomyopathy, spontaneous coronary artery dissection, cardiac chest pain, stress cardiomyopathy, scad management, scad

## Abstract

Spontaneous coronary artery dissection (SCAD) should be considered in the differential diagnosis of patients with provisional Takotsubo cardiomyopathy (TTS). However, because of overlapping clinical features, SCAD with subtle angiographic findings and wall motion abnormality like TTS can be easily missed. Therefore, our case highlights the importance of further investigation for SCAD.

## Introduction

Spontaneous coronary artery dissection (SCAD) is an essential consideration in the setting of myocardial infarction with nonobstructive coronary arteries (MINOCA) and stress cardiomyopathy (SCM), especially in young women. SCAD and stress cardiomyopathy (Takotsubo syndrome, TTS) may coexist. The diagnosis of SCAD requires a high index of suspicion, especially in the presence of seemingly normal coronary angiography, which may confound the diagnosis. Intravascular imaging is sensitive for subintimal hematoma, thus an important test when the diagnosis of SCAD is not apparent in an angiogram but is suspected clinically. In a patient with suspected TTS, the need for evaluation of coronary artery disease with cardiac computed tomography angiography (CTA) and coronary angiography is fundamental. TTS in SCAD is most probably triggered by ischemic insult.

This article was previously presented as a meeting abstract at the American College of Cardiology meeting held in March 2020.

## Case presentation

**Figure 1:**
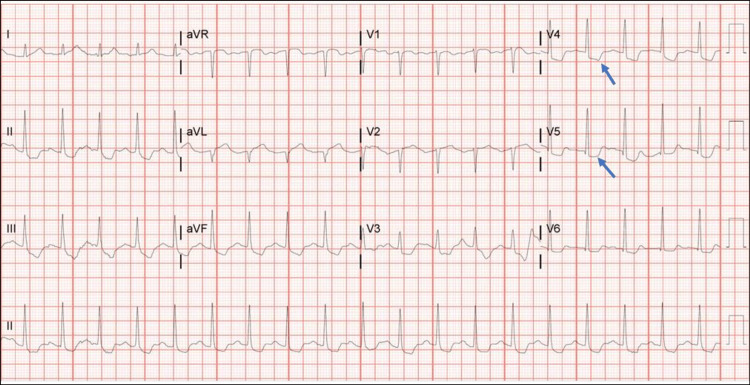
Initial electrocardiogram at presentation showing anterolateral ST depression. Initial electrocardiogram at presentation showed sinus tachycardia and diffuse inferior and anterolateral ST segment depression (blue arrows).

A 32-year-old African American woman was admitted following a witnessed cardiac arrest due to ventricular fibrillation at work. The patient had a spontaneous return of circulation after two defibrillation shocks and one round of cardiopulmonary resuscitation. The patient was unresponsive and intubated on arrival. She was admitted to the intensive care unit and started on hypothermia protocol. The patient developed cardiogenic shock 12 hours after admission and was placed on vasopressors. A 12-lead electrocardiogram (EKG) (Figure 1) showed diffuse inferior and anterolateral ST-segment depression.

Troponin peaked at 20 ng/mL (reference range = 0.0-0.04 ng/mL). CT of the chest showed no angiographic evidence of pulmonary embolism. The patient’s medical history included asthma, gastric ulcer, and ectopic pregnancy three months before admission.

Differential diagnosis

Differential diagnoses included ischemia from obstructive coronary disease (OCD), coronary vasospasm, acute coronary syndrome (ACS), and SCAD, whereas non-ischemic diagnoses included non-ischemic cardiomyopathy, idiopathic dilated cardiomyopathy, TTS, and drug-induced cardiomyopathy. Other potential differential diagnoses are sarcoidosis-related restrictive cardiomyopathy, arrhythmogenic right ventricular cardiomyopathy, and hypertrophic cardiomyopathy.

Investigations

**Figure 2:**
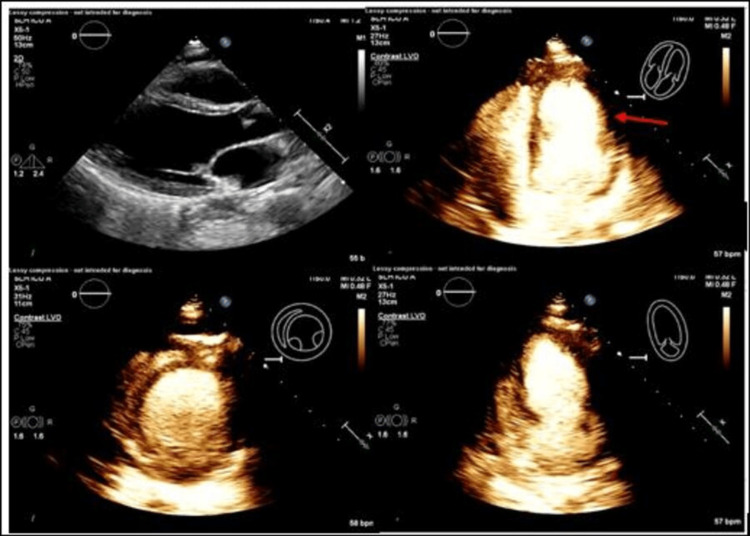
Echocardiogram with reduced ejection fraction concerning for stress cardiomyopathy. Echocardiogram revealed severely depressed left ventricular systolic function, with a calculated ejection fraction of 28% with apical and mid-anterior akinesis (red arrow) and preserved basal wall motion, suspicious of stress cardiomyopathy.

Echocardiogram (Echo) (Figure 2) showed left ventricular ejection fraction (LVEF) of 28% with apical and mid-anterior akinesis with preserved basal wall motion, suspicious of SCM.

Coronary angiography showed normal coronary arteries; however diagonal vessels were not clearly visible. A right heart catheterization showed low cardiac output (cardiac index = 1.68 L/m^2^, systemic vascular resistance = 2,247 dynes/cm^2^) and elevated filling pressures (pulmonary wedge pressure = 23 mmHg). An intra-aortic balloon pump was placed for support. The patient was off of mechanical ventilation within 24 hours, and the shock improved within 48 hours of hospitalization.

**Figure 3:**
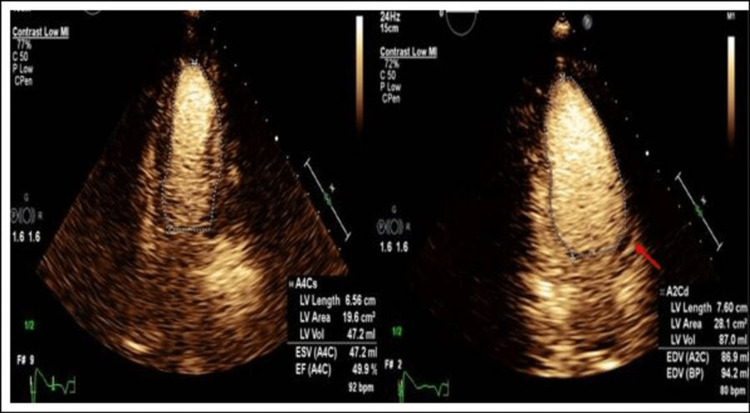
Follow-up echocardiogram with a significantly improved ejection fraction. Three days later, echocardiogram showed a remarkable improvement in overall wall motion abnormalities and an LVEF of 50%. Focal areas of akinesis involving the mid-anterolateral and mid-anterior segments (red arrow).

After recovery, the patient verbally excluded recent stressful events or a family history of sudden cardiac death. A repeat Echo on day four (Figure 3) showed an LVEF of 50%.

**Figure 4:**
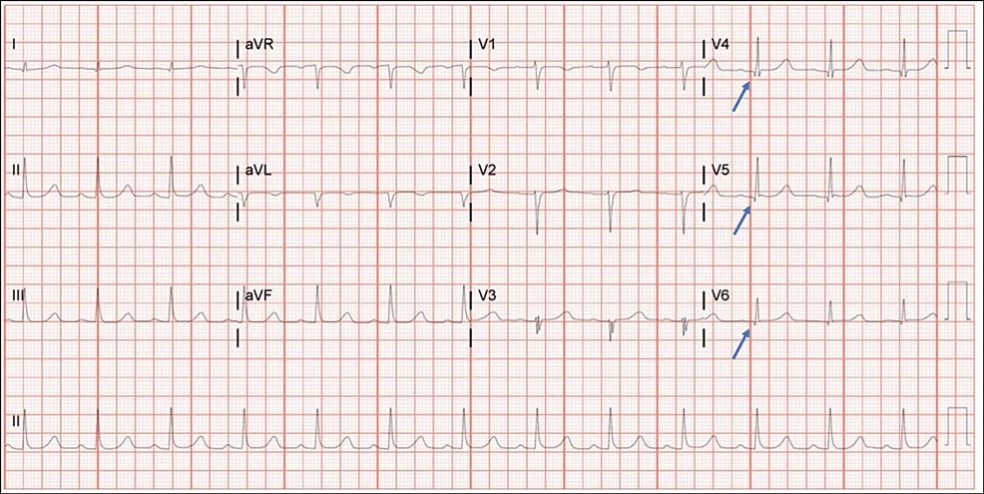
Day four electrocardiogram revealed an old anterolateral infarct. Day four electrocardiogram showing sinus rhythm, first-degree arteriovenous block, borderline right axis deviation, old anterolateral infarct (Q wave in v4, v5, and v6 pointed by blue arrows), and borderline prolonged QT interval.

Day four EKG showed sinus rhythm, first-degree arteriovenous block, borderline right axis deviation, old anterolateral infarct, and borderline prolonged QT interval (Figure 4).

Management

Given the early improvement in left ventricular (LV) function and lack of apparent OCD, our working diagnosis was SCM, a common etiology of MINOCA. A cardiac MRI, performed before placement of the subcutaneous implantable cardioverter-defibrillator, revealed anterior subendocardial late gadolinium enhancement, consistent with myocardial infarction. Other differential diagnoses included coronary embolism, vasospasm or dissection, and sarcoidosis. A fluorodeoxyglucose-positron emission tomography suggested infarction, not sarcoidosis. The patient was discharged with optimal medical therapy (aspirin, clopidogrel, carvedilol, and atorvastatin) and scheduled for a repeat coronary angiogram in six weeks as diagonal vessels were not visualized on the initial angiogram. Intravascular imaging is an uncommon modality to confirm SCAD as usually it is apparent on angiography. Intravascular ultrasound of the left main, left anterior descending (LAD), and diagonal artery showed subintimal hematoma/hemorrhage, confirming the diagnosis of SCAD.

Follow-up

The patient is doing well a year later during follow-up. She has New York Heart Association class II heart failure symptoms and is on furosemide, carvedilol, and aspirin.

## Discussion

SCAD is defined as epicardial coronary artery dissection without association with atherosclerosis, trauma, or iatrogenic [1]. It is characterized by the spontaneous formation of intramural hematoma within the coronary vessel wall. Two theories have been proposed on intramural hematoma. The first theory proposes that the primary pathological event is an intimal tear, while another theory proposes spontaneous hemorrhage from the vasa vasorum. This compressed true lumen leads to myocardial infarction (MI). The true prevalence of SCAD is not known yet because it is commonly underrecognized or missed diagnosed as TTS sometimes [1,2].

TTS is characterized by transient left ventricular wall motion abnormalities (LVWMA), not restricted to one coronary artery territory. Characteristically, there is a ballooning of the LV apex with systolic dysfunction that recovers fully over one to four weeks [2]. Both SCAD and TTS have a predilection for women and may be preceded by emotional or physical stress. Eventually, they both heal with a complete angiographic resolution of the dissected vessel and LV abnormality, respectively [1]. SCAD and TTS have a lot of similarities, including a presentation with MI, association with precipitating stressors, and reversal of WMA. A potential difference between the two conditions is that WMA resulting from SCAD corresponds to the affected artery and may not have the classic apical ballooning appearance of TTS if the distal LAD artery is unaffected. In SCAD, angiographic findings are acute and dynamic with resolution over time, whereas in TTS, angiographic abnormalities usually remain unchanged from previous studies [2].

There are several angiographic appearances of SCAD [1]. Given the similarities, differentiating between these two clinical entities oftentimes presents a diagnostic challenge. One theory suggests that SCAD may represent the precipitating event that ultimately results in TTS [3]. Another theory proposes that the classic LVWMA in TTS creates torsional and mechanical forces that ultimately result in a coronary artery dissection in an individual with an underlying predisposition [4].

The LVWMA has been theorized to arise from ischemia secondary to SCAD, especially in case presentations involving a wrap-around LAD artery [5]. The presence of coronary artery tortuosity may also lead to increased shear stress and ultimately lead to vascular vulnerability. There have been speculations that the coronary arteries traversing the anterior or anterolateral wall may be more vulnerable to spontaneous dissection as this region marks the transition point of the hyperdynamic basal segment and the remaining dyskinetic or akinetic LV segments. Vigorous contraction of the LV base results in excessive movement of the epicardial vessel and increases the shear stress on the vessel wall, which may ultimately lead to dissection. This is supported by the observation that 91.7% of the TTS patients in an observational study with the SCAD registry by Duran et al. had a dissected LAD or one of its branches [6]. However, LV ballooning has also been observed in SCAD cases without a long wrap-around LAD, as well as in cases in which solely SCAD-induced ischemia cannot fully explain the LVWMA.

Diagnosis of TTS and SCAD

Given the inability to distinguish coronary plaque rupture from coronary dissection by clinical presentation alone, these patients should be appropriately referred for urgent CA. If coronary artery obstruction is absent in the presence of elevated troponin, the patient’s syndrome is consistent with MINOCA for which the differential diagnosis includes TTS, SCAD, coronary vasospasm, coronary embolus, plaque erosion, and myocarditis [7]. Echo can provide important clues to direct further evaluation. Coronary CTA and intracoronary imaging should be considered as the next diagnostic step in patients for whom clinical suspicion remains high despite negative angiography [8].

Treatment

Conservative management should be considered in clinically stable patients without high-risk anatomy. Extended inpatient monitoring for three to five days is recommended. In patients with ongoing ischemia, left main artery dissection, or hemodynamic instability, urgent intervention with percutaneous coronary intervention (PCI) or coronary artery bypass grafting (CABG) can be considered [1]. CABG is appropriate for left main and proximal dissections, PCI complications, or ongoing ischemia. Systemic anticoagulation with heparin should be avoided after the diagnosis of SCAD. Dual antiplatelet therapy is recommended after PCI. In patients managed medically, most experts recommend aspirin for at least a year, and some also recommend clopidogrel for a few months to a year. Statin therapy is not recommended routinely and should be used if the patient has other indications [1]. Although the yield is low, referral to a genetic counselor with experience in arteriopathies should be considered. Extracoronary imaging from the brain to the pelvis that includes extracranial carotid arteries and renal arteries is important given the high co-prevalence with fibromuscular dysplasia [1].

## Conclusions

SCAD is an essential consideration in the setting of MINOCA and/or SCM, especially in young women. The diagnosis of SCAD requires a high index of suspicion, especially in the presence of seemingly normal CA, which may confound the diagnosis. Stress-induced cardiomyopathy (TTS) and SCAD have been shown to coexist. Coronary angiography and cardiac CTA are essential for evaluating coronary artery disease in patients with suspected TTS. TTS is most likely to be precipitated by ischemia in SCAD. Vascular imaging with intravascular ultrasound or optical coherence tomography is sensitive for subintimal hematoma, thus an important test when the diagnosis of SCAD is not apparent on an angiogram but is suspected clinically.
